# Development of Propofol-Encapsulated Liposomes and the Effect of Intranasal Administration on Bioavailability in Rabbits

**DOI:** 10.3390/pharmaceutics17111446

**Published:** 2025-11-09

**Authors:** Hitomi Ujita, Hitoshi Higuchi, Yukiko Nishioka, Saki Miyake, Riko Sato, Takuya Miyawaki

**Affiliations:** 1 Department of Dental Anesthesiology, Okayama University Hospital, 2-5-1 Shikata-cho, Kita-ku, Okayama 700-8525, Japanhiguti@md.okayama-u.ac.jp (H.H.); de421038@s.okayama-u.ac.jp (Y.N.); 2 Department of Dental Anesthesiology and Special Care Dentistry, Okayama University Graduate of Medicine, Dentistry and Pharmaceutical Sciences, 2-5-1 Shikata-cho, Kita-ku, Okayama 700-8525, Japan; de20049@s.okayama-u.ac.jp (S.M.); de427024@s.okayama-u.ac.jp (R.S.)

**Keywords:** liposome, propofol, bioavailability, intranasal administration

## Abstract

**Background/Objectives:** Propofol is frequently used as an intravenous anesthetic and is rapidly metabolized. Therefore, if it could be administered non-invasively (e.g., orally) as premedication, it might hasten emergence from anesthesia, thereby improving patient safety. However, it undergoes extensive first-pass metabolism in the liver and intestines, limiting the route for premedication. We evaluated whether intranasal delivery of a propofol-encapsulated liposome solution improves systemic exposure and bioavailability in rabbits. **Methods:** A propofol-encapsulated liposome solution was administered to rabbits via the intravenous, oral, and intranasal routes. Blood propofol concentrations were measured for up to 60 min after administration and the area under the concentration–time curve (AUC_0–60_) and bioavailability of the propofol-encapsulated liposome solution were compared with those of the non-encapsulated propofol formulation. The differences were tested by two-way analysis of variance (ANOVA) with Šidák’s post hoc multiple-comparisons test and the Mann–Whitney test (α = 0.05). **Results:** The AUC_0–60_ for blood propofol concentrations after intravenous administration was significantly higher with the propofol-encapsulated liposome solution than with the non-encapsulated propofol formulation (3038.8 ± 661.5 vs. 1929.8 ± 58.2 ng·min/mL; *p* = 0.0286). By contrast, no increase in blood propofol concentrations was observed after oral administration, whereas intranasal administration increased blood propofol concentrations and yielded significantly higher bioavailability compared with the non-encapsulated propofol formulation (16.4 ± 7.3% vs. 2.0 ± 1.2%; *p* = 0.0286). **Conclusions:** The findings of the present study suggest that intranasal liposomal propofol increased systemic availability compared with a non-encapsulated formulation, supporting further evaluation as a candidate premedication approach for propofol.

## 1. Introduction

Intranasal delivery to the central nervous system (CNS) using nanocarriers has been applied for neurological disorders such as Alzheimer’s disease [1,2,3], epilepsy [4,5], and Parkinson’s disease [6]. Various types of nanocarriers and delivery devices—each with distinct characteristics—have been reviewed [2,7,8,9]. Intranasal administration has been applied not only to therapeutics for neurological disorders but also to vaccines [10]. Its advantages include its noninvasiveness, rapid drug absorption [11,12], and a pathway through the trigeminal and olfactory sensory nerve that bypasses the blood–brain barrier (BBB), enabling direct delivery to the CNS [1,13]. Furthermore, even when drugs are absorbed through the nasal mucosa and enter the bloodstream to cross the BBB, intranasal administration allows them to bypass first-pass metabolism in the gastrointestinal tract.

Anesthetics, including midazolam and propofol, are highly lipophilic at physiological pH and readily cross the BBB, where they act on the CNS [14,15]. They are generally administered intravenously for general anesthesia and sedation. However, noninvasive premedication is frequently required for pediatric and/or intellectually disabled patients to reduce preoperative anxiety, enabling calm entry into the operating room and facilitating smooth induction of anesthesia [16]. Oral administration of the midazolam is one such noninvasive premedication method [17]. While its sedative effect is adequate, the strong bitterness of midazolam makes oral administration difficult in children and patients with intellectual disabilities. Intranasal administration is a useful alternative; however, the low pH and high concentration of midazolam can irritate the nasal mucosa and cause adverse effects. Regardless of whether it is administered intranasally or orally, its relatively long duration of action may extend into the postoperative period and delay emergence from anesthesia [18]. Recently, intranasal administration of a sedative, dexmedetomidine, has been reported to be effective; however, relatively high doses are required, which can prolong time to awakening and similarly delay emergence from anesthesia.

On the other hand, propofol, an intravenous anesthetic, has a rapid onset and short duration of action, with a distribution half-life of 2–8 min [19]. In addition, its context-sensitive half-time is shorter than that of other anesthetics such as midazolam [20]. If propofol could be administered noninvasively (e.g., orally) as premedication, it might hasten emergence from anesthesia, thereby improving patient safety. However, because propofol undergoes extensive first-pass metabolism in the gastrointestinal tract [21], no noninvasive route is currently available.

Previously, we established a method for preparing an oral midazolam-encapsulated liposome solution to reduce its bitterness [22] and improve its oral bioavailability [23]. Liposomes are closed vesicles composed of a phospholipid bilayer, a constituent of biological membranes. They possess both hydrophobic and hydrophilic regions, enabling encapsulation of drugs regardless of their aqueous or lipid solubility [24]. In addition, liposomes exhibit excellent in vivo biocompatibility and biodegradability [24] and are widely used clinically as carriers for various drugs [25]. They are also considered suitable nanocarriers for intranasal delivery [3]. Based on these considerations, we devised a new premedication delivery method of encapsulating propofol in liposomes and administering it intranasally.

Thus, the purpose of the present study was to establish a method for preparing propofol-encapsulated liposomes, to characterize them (encapsulation efficiency and particle size), and to evaluate the effect of intranasal administration on bioavailability in rabbits and thus its potential as a premedication approach. To this end, a propofol-encapsulated liposome solution was administered via intranasal, intravenous, and oral routes, and propofol bioavailability was compared across these routes.

The present study cites the first author’s PhD thesis (Okayama University, Japan), written in Japanese [26].

## 2. Materials and Methods

### 2.1. Agents

L-α-phosphatidylcholine (PCHL, P4139, purity: 100%), cholesterol (C3045, purity: 99%), dimyristoylphosphatidylcholine (DMPC, P2663, purity: 100%), heptane (34873, purity: 99.4%), and propofol reference standard (PHR1663, Lot: LRAC6351; certified purity 98.6 ± 1.97%) were purchased from Sigma-Aldrich (St. Louis, MO, USA). Propofol for administration (formulation for animals, purity: 95–105%) was purchased from Mylan Inc. (Osaka, Japan). Potassium dihydrogen phosphate solution (purity: ≥99.5%) was obtained from Wako Pure Chemical Industries (Osaka, Japan). High-performance liquid chromatography (HPLC)-grade water, hydrochloric acid solution (0.1 mol/L), and thymol (used as the internal standard, IS, for HPLC quantification, purity: ≥98.0%) were purchased from Nacalai Tesque (Tokyo, Japan). Methanol (purity: 100%), chloroform (purity: ≥99.7%), acetonitrile (purity: 100%) and ascorbic acid (purity: 99.7%) were purchased from Kanto Chemical Co., Inc. (Tokyo, Japan). Isoflurane was purchased from AbbVie GK (Tokyo, Japan).

### 2.2. Preparation of Propofol-Encapsulated Liposomes

According to the method of our previous studies [22,23], PCHL, cholesterol, DMPC, and the non-encapsulated propofol formulation were dissolved in a chloroform/methanol solvent mixture (chloroform:methanol = 2:3) and mixed in a fluorine-coatd pear-shaped flask at a molar ratio of PCHL:cholesterol:DMPC:propofol = 1:1:0.1:1. The mixture was evaporated on a rotary evaporator at 45 °C to prepare a lipid film, which was then dried under vacuum pump for 1 h. A solution of 1 M hydrochloric acid, 0.1% (*w*/*v*) ascorbic acid solution, and HPLC-grade water was mixed and adjusted to pH 4.0 and added to the lipid film. The mixture was shaken in a water bath at 45 °C to hydrate the lipid film and form a liposome suspension. The particle-size distribution of liposomes was measured by dynamic light scattering using a Zetasizer Nano ZSP instrument (Malvern Instruments, Worcestershire, UK) at a detector angle of 175° and a temperature of 25 °C.

For observing propofol-encapsulated liposomes with negative stain, samples were prepared for transmission electron microscopy (TEM) by placing approximately 10 µL of the liposome solution on Formvar-coated, carbon-stabilized 150-mesh copper grids. After a brief incubation, excess liquid was removed with filter paper, and the sample was negatively stained with 2% (*w*/*v*) uranyl acetate. The stained grids were allowed to dry overnight before imaging. TEM imaging was performed using an H-7650 transmission electron microscope (Hitachi High-Tech Corp., Tokyo, Japan) operating at an accelerating voltage of 80 kV. Digital images were captured using the integrated camera system for subsequent analysis of morphology and structural details.

To evaluate encapsulation efficiency of the liposomes, the propofol-encapsulated liposome suspension was centrifuged at 15,000× *g* for 20 min at 4 °C. The supernatant containing unencapsulated drug was removed, and 0.5 mL of an aqueous mixture of 1 M hydrochloric acid, 0.1% (*w*/*v*) ascorbic acid solution, and HPLC-grade water were added to the pellet and mixed to resuspend the liposomes. The encapsulation efficiency of liposomes was calculated as EE% = C_e/C_0 × 100 (C_0 is the propofol concentration in the suspension before centrifugation, and C_e is the propofol concentration in the resuspended liposomes after centrifugation).

### 2.3. Measurement of Propofol Concentrations

#### 2.3.1. HPLC Conditions

Propofol concentrations in the solution were measured with an HPLC (Shimadzu Co., Tokyo, Japan), as described in our previous study [27]. The mobile phase consisted of 560 mL of water, 340 mL of 0.25 M potassium dihydrogen phosphate solution, and 1800 mL of acetonitrile. The flow rate was 1.0 mL/min, and separation was performed at 40 °C on a LiChrospher 100 RP-18(e) with 5 µm column (4.0 mm i.d. × 150 mm; Merck KCaA, Darmstadt, Germany). Detection was carried out with a fluorescence detector (RF-20A; Shimazu Co., Tokyo, Japan) at excitation/emission wavelengths of 270/310 nm, respectively.

#### 2.3.2. Establishment of the Calibration Curve and Measurement of Propofol Concentrations

To measure propofol concentrations for determining the efficiency of liposomal propofol encapsulation, the propofol stock solution was diluted with methanol to prepare calibration standards at 1.25, 2.5, 5, and 10 µg/mL. Each standard (0.2 mL) was combined with 2.2 mL of heptane for encapsulation-efficiency assays and then vortex-mixed for 1 min and centrifuged at 1500× *g* for 10 min to perform liquid–liquid extraction. From the organic phase, 600 µL of the supernatant was collected, and then 40 µL of thymol (IS, 5 µg/mL) and 50 µL of 0.1 M potassium dihydrogen phosphate were added. After vortex-mixing for 1 min and centrifugation at 1500× *g* for 10 min, the resulting supernatant (200 µL) was injected into an HPLC system for measuring propofol concentrations. For each concentration, the peak-area ratio of propofol to thymol (IS) was determined and used to construct a four-point calibration curve (1.25–10 µg/mL) by least-squares linear regression. The calibration curve was linear over 1.25–10 µg/mL (Figure S1; correlation coefficient > 0.999). The resulting regression equation was then used to calculate propofol concentrations from the standard’s peak-area ratios for determining liposomal encapsulation efficiency.

For measuring blood propofol concentrations, the propofol stock solution was diluted with methanol to prepare calibration standards at 9.4, 18.8, 37.5, and 150 ng/mL. Heptane (0.9 mL) was added to 0.2 mL of each standard; then, the mixture was vortex-mixed for 1 min and centrifuged at 1500× *g* for 10 min to perform liquid–liquid extraction. From the organic phase, 600 µL of the supernatant was collected, and then 40 µL of thymol (IS, 0.05 µg/mL) and 50 µL of 0.1 M potassium dihydrogen phosphate were added. After vortex-mixing for 1 min and centrifugation at 1500× *g* for 10 min, the resulting supernatant (200 µL) was injected into an HPLC system to measure propofol concentrations. As described above, peak-area ratios of propofol to thymol (IS) were obtained for each concentration and a calibration curve was generated by least-squares linear regression. The calibration was linear over 9.4–150 ng/mL (Figure S2; correlation coefficient > 0.999), and the resulting regression equation was used to calculate propofol concentrations from the standard’s peak-area ratios for measuring blood propofol concentrations. The propofol concentration detection limit was 7.6 ng/mL.

### 2.4. Animal Model

The experiments were conducted as described in our previous studies [6,7]. Twenty-four clean male New Zealand White rabbits, aged 10–11 weeks and weighing 1.90–2.40 kg (Japan SLC, Inc., Hamamatsu, Japan), were used for in vivo experiments. The experiments were approved by the Animal Experiment Management Committee of Okayama University (approval number: OKU-2022591) and carried out in strict accordance with the recommendations outlined in the Guide for the Care and Use of Laboratory Animals at Okayama University and the International Guiding Principles for Biomedical Research Involving Animals by the International Council for Laboratory Animal Science. The experiments were performed after a one-week acclimation period, during which rabbits were fed pellets and water in an animal room controlled at a temperature of 25 °C. The pellets were changed to AIN93M (Oriental Yeast, Tokyo, Japan), in which cellulose was replaced with alfalfa (dietary fiber: 30.0% or less), starting 3 days before the experiments, and the animals were given water ad libitum and fasted for 18 h prior to use.

### 2.5. In Vivo Experiments with Intravenous Administration

The in vivo experiments were conducted according to the methods described in our previous studies [6,7]. Eight rabbits were anesthetized with isoflurane, and a catheter was inserted into the femoral artery to continuously collect peripheral arterial blood samples. The non-encapsulated propofol formulation was diluted with a fat emulsion product (Intralipos Injection 10%, Otsuka Pharmaceutical Co., Ltd., Tokyo, Japan), which is identical to the lipid component of the non-encapsulated propofol formulation, and the propofol-encapsulated liposome solution was diluted with a mixture of ascorbic acid and hydrochloric acid (pH 4.0). The administered propofol dose in the test solutions was set to 1.0 mg/kg, and the volume was adjusted to 2.0 mL for intravenous administration.

After confirming the arousal of rabbits at least 60 min after completion of isoflurane-induced inhalation anesthesia—based on leg movement and eye opening—a bolus dose of each test solution was administered through the auricular vein. After administration, peripheral arterial blood (1.0 mL each) was collected from the femoral artery at 1, 3, 5, 10, 30, and 60 min. The blood samples were centrifuged at 1500× *g* for 10 min at room temperature, and the separated plasma samples were stored at −30 °C.

For preparation, heptane (900 µL) was added to each plasma sample (200 µL) and centrifuged at 1500× *g* for 10 min, and the heptane layer was collected. Thymol (IS, 0.05 µg/mL, 40 µL) and 0.1 M potassium dihydrogen phosphate solution (50 µL) were added to the collected layer (600 µL) and centrifuged at 1500× *g* for 10 min, and the resulting supernatant (200 µL) was injected into an HPLC. Propofol concentrations in the samples were calculated using the regression equation described above, and the area under the blood concentration–time curve from 0 to 60 min (AUC_0–60_) was calculated for each animal.

### 2.6. In Vivo Experiments with Oral Administration

Eight rabbits were prepared in the same manner as in the intravenous administration experiments described above. After femoral arterial catheterization, a 6.5 Fr stomach tube (target length, 14 cm) was inserted through the nose to administer the test solutions. The final tube length was determined by listening for air entry into the stomach by stethoscope. The administered propofol dose in the test solutions was set at 1.0 mg/kg, and the volume was adjusted to 10.0 mL for oral administration. After confirming arousal, the test solution was administered through the tube over 60 s, followed by 2.0 mL of saline over 10 s to expel the remaining solution. After administration, peripheral arterial blood (1.0 mL each) was collected from the femoral artery at 3, 5, 10, 30, and 60 min. As in the intravenous administration experiment, propofol concentrations in the samples were measured by HPLC, and AUC_0–60_ was calculated for each animal.

### 2.7. In Vivo Experiments with Intranasal Administration

Eight rabbits were prepared in the same manner as in the intravenous administration experiments described above. However, to prevent sneezing due to nasal stimulation, the test solution was administered into the nasal cavity using a 20G indwelling IV catheter (BD Angiocath, 20 G, 1.1 mm × 48 mm, Becton, Dickinson and Company, Franklin Lakes, NJ, U.S.A.) under 2.5–3.5% isoflurane anesthesia. Prior to this experiment, we confirmed that rabbits did not show any reactions other than sneezing when the test solution was administered intranasally. The dose of propofol in test solutions was set at 1.0 mg/kg, and the volume was adjusted to 1.4 mL for intranasal administration. Similarly to the oral administration experiment, peripheral arterial blood (1.0 mL each) was collected from the femoral artery at 3, 5, 10, 30, and 60 min; the propofol concentrations in the samples were measured by HPLC as described above; and AUC_0–60_ was calculated for each animal.

### 2.8. Statistical Analysis

In each experiment, changes in the blood propofol concentration and AUC_0–60_ were compared between non-encapsulated propofol formulation and propofol-encapsulated liposome solution. The significance of differences in blood propofol concentrations was evaluated by two-way analysis of variance (ANOVA) followed by post hoc Šidák’s multiple-comparison test, and differences in AUC_0–60_ were evaluated by the Mann–Whitney test using GraphPad Prism (Version 4.0c; GraphPad Software, San Diego, CA, USA). *p* < 0.05 was considered statistically significant. Data are expressed as mean ± standard deviation (SD).

## 3. Results

### 3.1. Characteristics of Propofol-Encapsulated Liposomes

**Figure 1:**
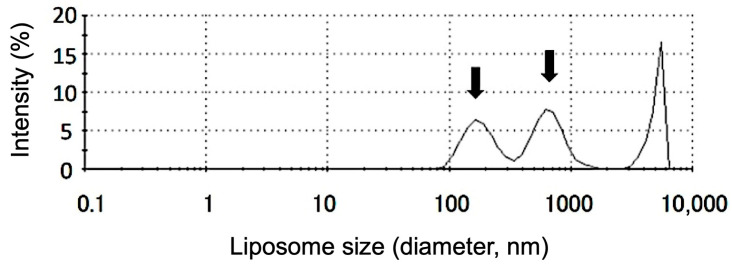
Typical size-distribution of the liposome particles in propofol-encapsulated liposome solution. This figure shows the particle size distribution of the prepared propofol-encapsulated liposomes, measured by dynamic light scattering using a Zetasizer Nano ZSP. The vertical axis represents fluorescence intensity, and the horizontal axis represents particle diameter. The size distribution of liposome particles exhibited two peaks, with ranges of 124.9–183.3 and 418.7–675.0 nm in diameter (*n* = 4). The arrows indicate the peak positions.

The propofol encapsulation efficiency of the liposomes was 38.74 ± 7.96% (29.44–47.23%) (Table S1). The loading capacity was 0.121 ± 0.023 mol/mol. Figure 1 shows the typical size distribution of liposome particles in the propofol-encapsulated liposome solution. The size distribution of liposome particles exhibited two intensity-weighted peaks, with ranges of 124.9–183.3 and 418.7–675.0 nm in diameter. The zeta potential of the liposomes was −27.2 ± 0.83 mV.

**Figure 2:**
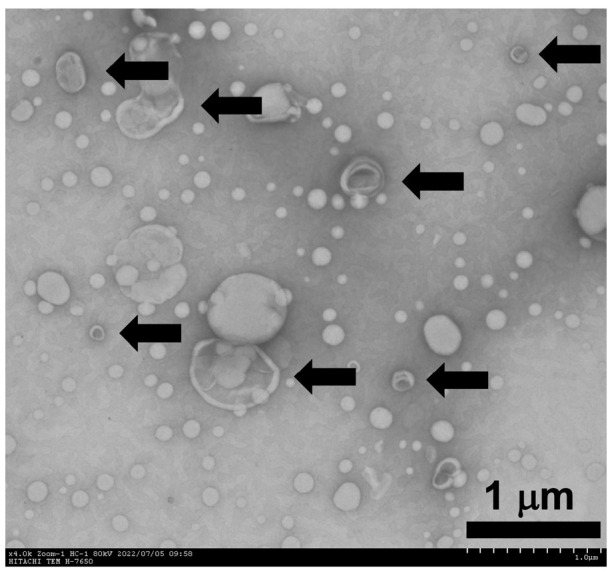
TEM image of propofol-encapsulated liposomes. The image was prepared by negative staining with 2% (*w*/*v*) uranyl acetate and imaged at 80 kV. Two particle-size populations are evident, consistent with the particle-size distribution (Figure 1). Arrows indicate representative vesicles with phospholipid bilayer contrast. Scale bar: 1 µm.

Captured digital TEM images of propofol-encapsulated liposomes showed two particle-size populations, as shown in the size distribution above (Figure 2).

### 3.2. Blood Concentrations After Intravenous Administration of the Test Solutions

**Figure 3:**
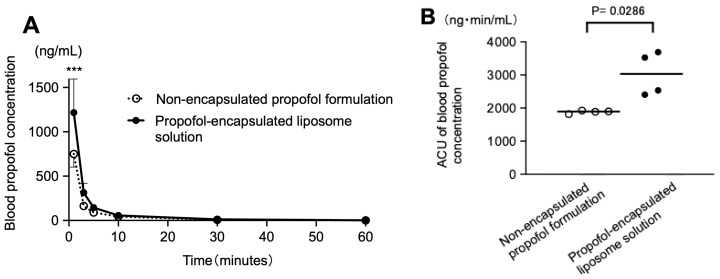
Blood propofol concentrations after intravenous administration of the test solutions (non-encapsulated propofol formulation and propofol-encapsulated liposome solution; 4 rabbits per group). The propofol dose in the test solutions was set to 1.0 mg/kg, and the volume was adjusted to 2.0 mL for intravenous administration. (**A**): Time courses of the changes in the blood propofol concentration (ng/mL). (**B**): Area under the blood concentration–time curve from 0 to 60 min (min) (AUC_0–60_, ng·min/mL) for the test solutions. The AUC_0–60_ for the propofol-encapsulated liposome solution was significantly higher than that of the non-encapsulated propofol formulation. *** *p* < 0.0001 vs. non-encapsulated propofol formulation.

Figure 3A and Table S2 show the time courses of blood propofol concentrations after intravenous administration of the test solutions (non-encapsulated propofol formulation vs. propofol-encapsulated liposome solution). At 1 min post-dose, blood propofol concentrations after injection of the propofol-encapsulated liposome solution were significantly higher than those after injection of the non-encapsulated propofol formulation (*p* < 0.0001; propofol-encapsulated liposome solution vs. non-encapsulated propofol formulation; two-way ANOVA followed by post hoc Šidák’s multiple-comparison test). Figure 3B and Table S3 show the AUC_0–60_ for the test solutions. The AUC_0–60_ for the propofol-encapsulated liposome solution was significantly higher than that of the non-encapsulated propofol formulation (3038.8 ± 661.5 vs. 1929.8 ± 58.2 ng·min/mL; *n* = 4 each; *p* = 0.0286; propofol-encapsulated liposome solution vs. non-encapsulated propofol formulation; Mann–Whitney test).

### 3.3. Blood Concentrations After Oral Administration of the Test Solutions

Blood propofol concentrations after oral administration of the test solutions (non-encapsulated propofol formulation and propofol-encapsulated liposome solution) were below the HPLC limit of detection at all time points. Therefore, AUC_0–60_ was zero in both groups.

### 3.4. Blood Concentrations After Intranasal Administration of the Test Solutions

**Figure 4:**
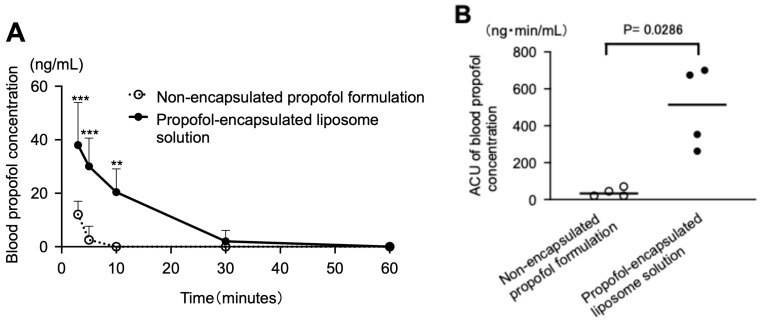
Blood propofol concentrations after intranasal administration of the test solutions (non-encapsulated propofol formulation and propofol-encapsulated liposome solution; 4 rabbits per group). The propofol dose in the test solutions was set to 1.0 mg/kg, and the volume was adjusted to 1.4 mL for intranasal administration. (**A**): Time courses of the changes in the blood propofol concentration (ng/mL). (**B**): Area under the blood concentration-time curve from 0 to 60 min (min) (AUC_0–60_, ng·min/mL) for the test solutions. The AUC_0–60_ of the propofol-encapsulated liposome solution was significantly higher than that of the non-encapsulated propofol formulation. *** *p* < 0.0001, ** *p* < 0.01 vs. non-encapsulated propofol formulation.

Figure 4A and Table S4 show the time courses of blood propofol concentrations after intranasal administration of the test solutions (non-encapsulated propofol formulation and propofol-encapsulated liposome solution). At 3, 5, and, 10 min post-dose, concentrations after administration of the propofol-encapsulated liposome solution were significantly higher than those after administration of the non-encapsulated propofol formulation (*p* < 0.0001 at 3 and 5 min; *p* = 0.0017 at 10 min; propofol-encapsulated liposome solution vs. non-encapsulated propofol formulation; two-way ANOVA followed by post hoc Šidák’s multiple-comparison test). Figure 4B and Table S5 show the AUC_0–60_ for the test solutions. The AUC_0–60_ of the propofol-encapsulated liposome solution was significantly higher than that of the non-encapsulated propofol formulation (497.5 ± 222.5 vs. 39.2 ± 23.9; *n* = 4 each; *p* = 0.0286; propofol-encapsulated liposome solution vs. non-encapsulated propofol formulation; Mann–Whitney test). Furthermore, the bioavailability of the propofol-encapsulated liposome solution and the non-encapsulated propofol formulation was 16.4 ± 7.3% vs. 2.0 ± 1.2%, respectively (Table S6). Therefore, intranasal bioavailability of the propofol-encapsulated liposome solution was significantly higher than that of the non-encapsulated propofol formulation (*p* = 0.0286; propofol-encapsulated liposome solution vs. non-encapsulated propofol formulation; *n* = 4 each; Mann–Whitney test).

## 4. Discussion

Propofol has very low aqueous solubility owing to its aromatic ring and isopropyl groups [28]. Common strategies to solubilize poorly water-soluble drugs in aqueous media include pH adjustment, the use of surfactants, and formulation as fat emulsions. The commercial formulation of propofol is a 1% (*w*/*v*) propofol oil-in-water emulsion, containing 10% soybean oil, 2.25% glycerol, and 1.2% purified egg phosphatide [29]. The triglyceride component of the emulsion is hydrolyzed by lipoprotein lipase on the capillary endothelium in the circulation, thereby promoting the release of propofol from the emulsion phase [30]. Metabolism of propofol primarily involves hepatic glucuronidation and hydroxylation followed by glucuronidation and sulfation by cytochrome P450 (CYP) [31]. Approximately 70% of propofol is conjugated to propofol glucuronide by uridine 5′-diphosphate (UDP) glucuronyltransferase [32]. The remaining approximately 29% of propofol is hydroxylated to form 2,6-diisopropyl-1,4-quinol [32]. This step involves numerous different CYP isoforms, with CYP2B6 and CYP2C9 as the primary catalysts [32]. The resulting 2,6-Diisopropyl-1,4-quinol is subsequently conjugated to 2,6-diisopropyl-1,4-quinol-4-sulfate, 2,6-diisopropyl-1,4-quinol-1-glucuronide, and 2,6-diisopropyl-1,4-quinol-4-glucuronide, and is excreted in urine [33]. These metabolites lack sedative activity [32]. However, evidence for extrahepatic metabolism includes the detection of propofol glucuronide in urine after intravenous administration during the anhepatic phase of liver transplantation [34,35]. Furthermore, systemic clearance of propofol decreases by about 42% during the anhepatic phase [35], further supporting extrahepatic metabolism. Although the responsible organ(s) remain unidentified, propofol glucuronidation occurs in the liver, kidneys, and small intestine [36]. Thus, the kidneys and small intestine are considered to contribute to extrahepatic metabolism.

In the present study, we established a method for preparing propofol-encapsulated liposomes. The liposomes we prepared in the present study are unmodified and their compositions are already used in many formulations in clinical practice [10], indicating there are no safety concerns to consider. Regarding stability of the liposomes, our previous research [22] demonstrated liposomal stability in liposomes with a similar composition and a different drug incubated in the near-physiological Tris-hydrochloride buffer and maintained at >80% encapsulation for one week. However, the liposomes prepared in the present study exhibited a bimodal particle-size distribution and could not be purified to a relatively small, monodisperse population. This trend was also observed with another drug [23], suggesting that it may be lipid-dependent. This phenomenon may be attributable to physicochemical properties specific to propofol. We attempted fine granulation in preliminary experiments; however, the recovery yield decreased further, and we were unable to obtain sufficient material for administration in rabbits. Therefore, although the pre-granulation liposome solution was used in the present study, fine granulation of the liposomes should be explored in future research.

It has been reported that adjusting the microparticle size to 100 nm improves absorption efficiency in the intestinal mucosa [37,38]. Furthermore, when the particle size is 50 nm or smaller, liposomes may nonspecifically penetrate blood-vessel walls during circulation [39]. Therefore, the optimal liposome diameter is considered to be 50–100 nm. Furthermore, the zeta potential of the liposomes prepared in the present study was negative (−27.2 ± 0.83 mV). Anionic liposomes are generally considered safer than cationic ones [40]. PEGylation can increase liposome stability and transport across the mucosal membrane and BBB [7]. However, our previous studies [23] showed that PEGylation induces liposome aggregation and reduces mucosal permeability in oral administration. Accordingly, we did not apply PEGylation in the present study, as it might similarly diminish intranasal permeability. However, a modest PEGylation or chitosan coating may be required to improve mucosal permeability.

When propofol-encapsulated liposome solution was administered intravenously, blood propofol concentrations at 1 min were significantly higher than those after injection of the non-encapsulated propofol formulation, and the AUC_0–60_ with the administration of propofol-encapsulated liposome solution was higher than that with the non-encapsulated propofol formulation. Encapsulation of propofol in liposomes may have temporarily avoided hepatic metabolism immediately after administration. Alternatively, the encapsulation of propofol may have affected extrahepatic metabolism, resulting in a short-term increase in the blood concentrations [23]. On the other hand, it has been reported that, when propofol is administered orally, the first-pass effect occurs most prominently in the intestinal mucosa [21]. Approximately 80% of administered propofol undergoes first-pass metabolism. Therefore, orally administered propofol has generally been considered unlikely to increase blood concentrations or produce clinical effects, which is consistent with the results of the present study.

In general, nasal administration allows drugs to enter the bloodstream directly, and the large surface area, high permeability, and vascularization of the nasal mucosa allow high systemic absorption [41]. Furthermore, hepatic first-pass metabolism can be avoided, resulting in a faster onset of action and higher bioavailability compared with oral administration [41]. In addition, it has been reported that the nasal mucosa absorbs lipophilic drugs more readily than hydrophilic drugs [42]. Intranasal administration of propofol formulation showed little increase in blood concentration. Ex vivo studies have reported that propofol emulsions barely penetrate the nasal mucosa [43] This is attributed to the slightly larger size of propofol emulsions in lipid preparations (>200 nm in diameter) [44]. However, because the formulation contains a small amount of free propofol [44], this is thought to be absorbed through the mucosa, causing a slight increase in blood concentration.

A liposomal formulation of rivastigmine has been shown to prevent drug degradation in the nasal cavity and to carry the drug through the mucosal barriers [45]. It has been reported that relatively small liposomes are absorbed directly and carried into circulation. Liposomes have been reported to cross or be transported across through the BBB [7]. Liposomes have been reported to be endocytosed by olfactory epithelial cells, with subsequent intracellular release. Furthermore, propofol that leaks from liposomes or is released upon liposome breakdown can be absorbed through the nasal mucosal capillaries and transported into the circulation, reaching the CNS by crossing the BBB [46]. In addition, propofol is considered to reach the CNS via the trigeminal or olfactory pathways without crossing the BBB.

The present study has several limitations. Firstly, rabbits were used in the experiments, because frequent arterial blood sampling over 1 h was required, necessitating the use of relatively large animals. In addition, it is known that glucuronidation occurs in the rabbit liver, similar to that in humans [47]. Therefore, rabbits were considered suitable for the present experiments. Following oral administration, the drug is absorbed from the gastrointestinal tract—primarily in the small intestine—into the capillaries and passes through the portal vein to the liver. However, the anatomy of the rabbit nasal cavity differs from that of humans. Absorption pathways also vary across nasal regions [2]. Furthermore, the use of a relatively long catheter for intranasal administration—intended to prevent leakage from the nasal cavity—may have allowed propofol to flow into the pharynx or esophagus, resulting in low bioavailability. As a next step, once sufficient evidence has been established in this animal model, these issues should be addressed in clinical studies.

Secondly, when using propofol for general anesthesia in humans, a dose of 1.0–2.5 mg/kg induces the required anesthetic effect [29,48]. Therefore, the administered dose of propofol in the present study was set at 1.0 mg/kg, and the same dose was used for both intranasal and oral administration. However, the propofol concentration after intranasal administration of the propofol-encapsulated liposome solution was not high enough to induce an appropriate anesthetic effect in the present study. For clinical use, higher propofol encapsulation efficiency in liposomes and greater bioavailability will be required. However, another property of propofol is that it is absorbed by not only plastic but also glass laboratory equipment. Therefore, the inner surface of the pear-shaped glass flask used to prepare lipid membranes was coated with a fluoropolymer to minimize adsorption to the glass surface. Furthermore, oxidation appeared to increase propofol’s tendency to adsorb to glass surfaces. Therefore, in the present study, the propofol stock solution was stored under 100% nitrogen (N2) at −20 °C. In addition, ascorbic acid (an antioxidant) was added to the liposome suspension, as described in a previous report [49]. Further improvements to the encapsulation method will be needed to enhance encapsulation efficiency.

Thirdly, when drugs are administered intranasally in a conscious state, the formulation pH may irritate the nasal mucosa [50], and the amount that can be administered is limited. In the present study, rabbits were given isoflurane, an inhalational anesthetic, to induce mild sedation, and intranasal administration was then performed. In a preliminary experiment, we attempted to administer the test solutions intranasally to a rabbit without anesthesia. The rabbit sneezed but showed no signs of distress, which was consistent with the response to physiological saline. However, because sneezing impeded full dosing, we administered the test solutions under isoflurane anesthesia. However, the discomfort caused by intranasal administration of the propofol-encapsulated liposome solution remains unclear in an animal model, and further investigation is needed to determine the appropriateness of this method. Authors should discuss the results and how they can be interpreted from the perspective of previous studies and the working hypotheses. The findings and their implications should be discussed in the broadest context possible. Future research directions may also be highlighted.

## 5. Conclusions

In the present study, we successfully encapsulated propofol with liposomes and prepared propofol-encapsulated liposomes with an encapsulation efficiency of approximately 30%. The propofol-encapsulated liposome solution was administered to rabbits at a dose equivalent to 1.0 mg/kg of propofol (as propofol) via the intravenous, oral, and intranasal routes. Following intravenous administration, AUC_0–60_ for blood propofol concentrations was significantly higher with the propofol-encapsulated liposome solution than with the non-encapsulated propofol formulation. On the other hand, no increase in the blood concentrations was observed after oral administration, whereas intranasal administration led to significantly higher blood propofol concentrations of propofol, AUC_0–60_, and bioavailability compared with the non-encapsulated propofol formulation. These results indicate that the intranasal bioavailability of the propofol-encapsulated liposome solution is higher than that of the non-encapsulated propofol formulation. Although further improvements are necessary before the propofol-encapsulated liposome solution can be used clinically, the findings of the present study suggest that intranasal administration of propofol may be a new premedication method to relieve preoperative anxiety in children and patients with intellectual disabilities, thereby hastening emergence from anesthesia and improving patient safety.

## Data Availability

The original contributions presented in this study are included in the article/Supplementary Materials. Further inquiries can be directed to the corresponding author.

## Supplementary Materials

The following supporting information can be downloaded at: https://www.mdpi.com/article/10.3390/pharmaceutics17111446/s1, Figure S1: Calibration curve for propofol over the range 1.25–10 µg/mL, Figure S2: Calibration curve for propofol over the range 9.4–150 ng/mL, Table S1: Propofol encapsulation efficiency, Table S2: Blood propofol concentrations after intravenous administration of the test solutions, Table S3: AUC_0-60_ for the test solutions after intravenous administration, Table S4: Blood propofol concentrations after intranasal administration of the test solutions, Table S5: AUC_0-60_ for the test solutions after intranasal administration, Table S6: Bioavailability for the test solutions after intranasal administration.

## Abbreviations

The following abbreviations are used in this manuscript:

AUC_0–60_	area under the blood concentration-time curve from 0 to 60 min
ANOVA	analysis of variance
CNS	central nervous system
BBB	blood–brain barrier
PCHL	L-α-phosphatidylcholine
DMPC	dimyristoylphosphatidylcholine
HPLC	high-performance liquid chromatography
TEM	transmission electron microscopy
IS	internal standard
