# Supplementary figures and images for: Development of Propofol-Encapsulated Liposomes and the Effect of Intranasal Administration on Bioavailability in Rabbits
Source: Pharmaceutics. 2025 Nov 9;17(11):1446. doi: 10.3390/pharmaceutics17111446 (PMC12655750; doi:10.3390/pharmaceutics17111446)

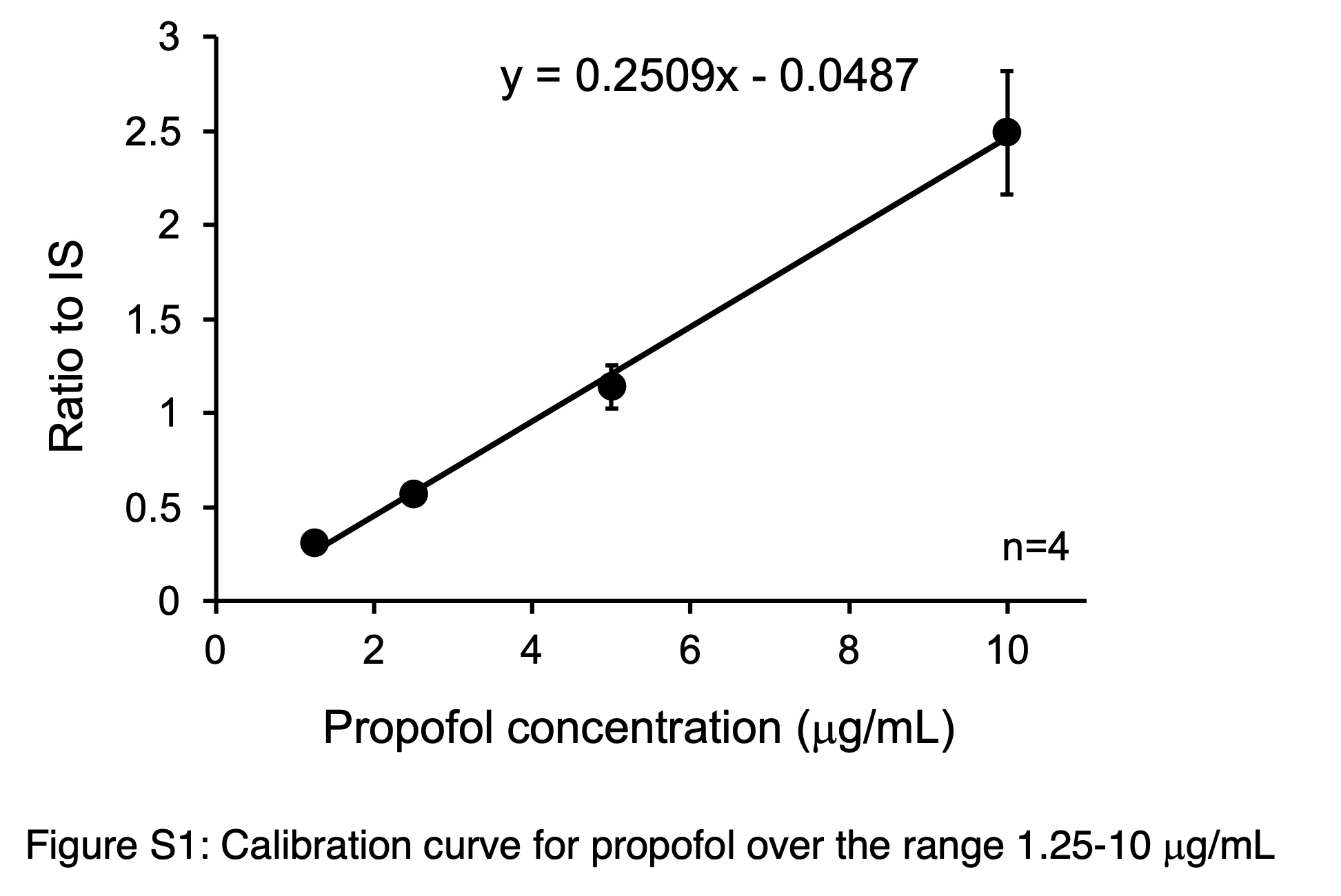

Supplement: Supplementary file 1 [file pharmaceutics-17-01446-s001.zip › Figure S1.tiff]

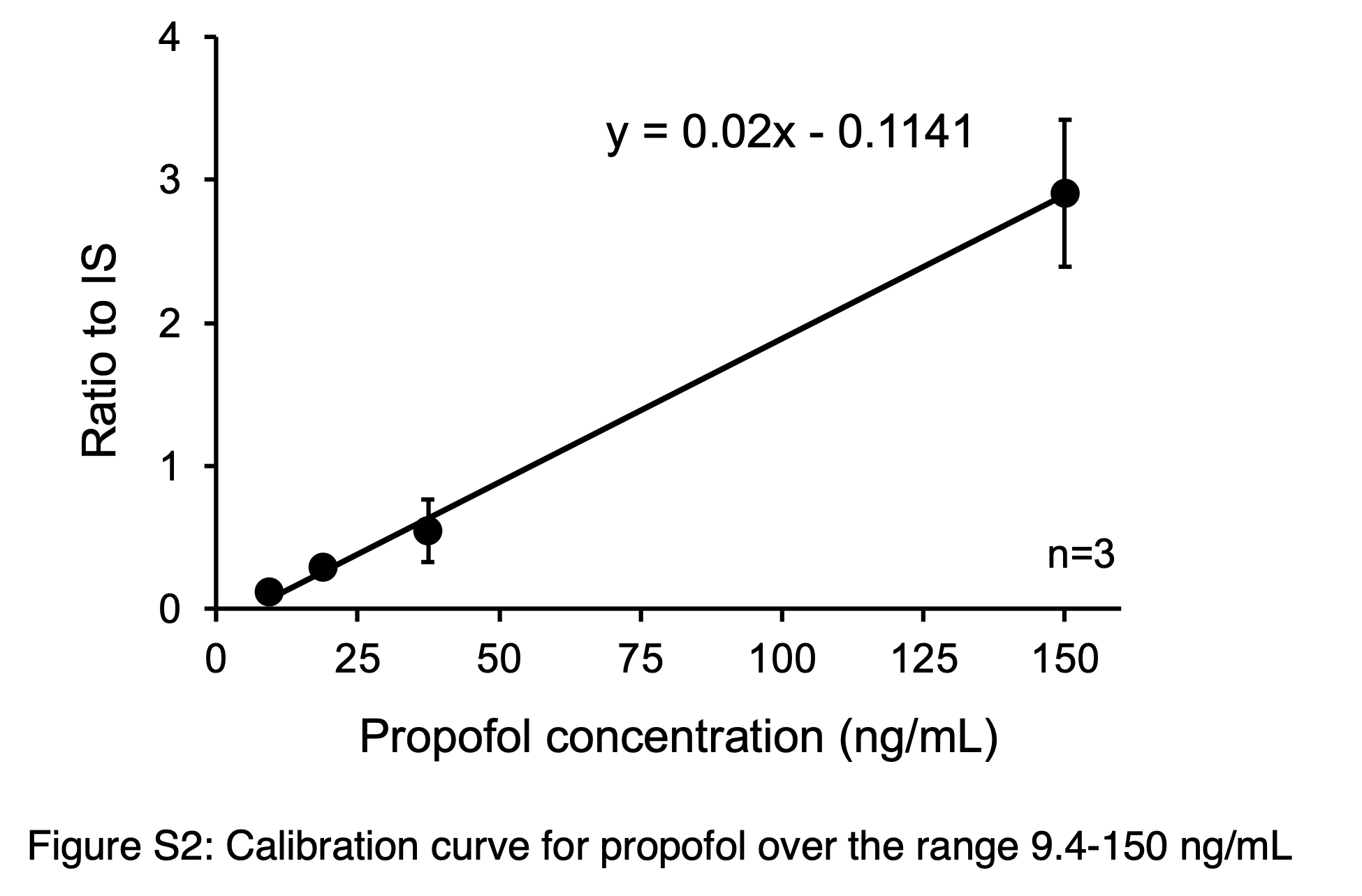

Supplement: Supplementary file 1 [file pharmaceutics-17-01446-s001.zip › Figure S2.tiff]
